# Histopathological Analysis of Tumor Microenvironment and Angiogenesis in Pheochromocytoma

**DOI:** 10.3389/fendo.2020.587779

**Published:** 2020-11-10

**Authors:** Xin Gao, Yuto Yamazaki, Alessio Pecori, Yuta Tezuka, Yoshikiyo Ono, Kei Omata, Ryo Morimoto, Yasuhiro Nakamura, Fumitoshi Satoh, Hironobu Sasano

**Affiliations:** ^1^ Department of Pathology, Tohoku University Graduate School of Medicine, Sendai, Japan; ^2^ Division of Clinical Hypertension, Endocrinology and Metabolism, Tohoku University Graduate School of Medicine, Sendai, Japan; ^3^ Division of Nephrology, Endocrinology, and Vascular Medicine, Tohoku University Hospital, Sendai, Japan; ^4^ Division of Pathology, Faculty of Medicine, Tohoku Medical and Pharmaceutical University, Sendai, Japan

**Keywords:** pheochromocytoma, pathology, immunohistochemistry, tumor microenvironment, angiogenesis

## Abstract

Pheochromocytomas (PHEOs) are relatively rare catecholamine-producing tumors derived from adrenal medulla. Tumor microenvironment (TME) including neoangiogenesis has been explored in many human neoplasms but not necessarily in PHEOs. Therefore, in this study, we examined tumor infiltrating lymphocytes (CD4 and CD8), tumor associated macrophages (CD68 and CD163), sustentacular cells (S100p), and angiogenic markers (CD31 and areas of intratumoral hemorrhage) in 39 cases of PHEOs in the quantitative fashion. We then compared the results with pheochromocytoma of the adrenal gland scaled score (PASS), grading system for pheochromocytoma and paraganglioma (GAPP) and the status of intra-tumoral catecholamine-synthesizing enzymes (TH, DDC, and PNMT) as well as their clinicopathological factors. Intratumoral CD8 (p = 0.0256), CD31 (p = 0.0400), and PNMT (p = 0.0498) status was significantly higher in PHEOs with PASS <4 than PASS ≧4. In addition, intratumoral CD8^+^ lymphocytes were also significantly more abundant in well-than moderately differentiated PHEO according to GAPP score (p = 0.0108) and inversely correlated with tumor size (p = 0.0257). Intratumoral CD68^+^ cells were significantly higher in PHEOs with regular or normal histological patterns than those not (p = 0.0370) and inversely correlated with tumor size (p = 0.0457). The status of CD163 was significantly positively correlated with that of CD8 positive cells (p = 0.0032). The proportion of intratumoral hemorrhage areas was significantly higher in PHEOs with PASS ≧4 (p = 0.0172). DDC immunoreactivity in tumor cells was significantly positively correlated with PASS score (p = 0.0356) and TH status was significantly higher in PHEOs harboring normal histological patterns (p = 0.0236) and cellular monotony (p = 0.0219) than those not. Results of our present study did demonstrate that abundant CD8^+^ and CD68^+^ cells could represent a histologically low-scored tumor. In particular, PHEOs with increased intratumoral hemorrhage should be considered rather malignant. In addition, abnormal catecholamine-producing status of tumor cells such as deficient PNMT and TH and increased DDC could also represent more aggressive PHEOs.

## Introduction

Pheochromocytomas (PHEOs) and paragangliomas (PGLs) are relatively rare tumors originating from the adrenal medulla at proximately 2 to 9.1 per 1 million adults frequently associated with cardiovascular complications due to excessive catecholamine production (1–3). The difference between PHEOs and PGLs depends on the primary sites, *i.e.*, intra- or extra-adrenal glands (4), and the former one termed as PHEOs (5). It is generally extremely difficult to differentiate benign from malignant PHEOs based upon clinical or even histopathological findings, and all PHEOs are currently considered potentially malignant tumor in the WHO 2017 classification (6). However, toward establishing more accurate histopathological diagnosis of PHEOs, Thompson et al., proposed a novel scoring system for the patients with PHEOs, *i.e.*, PASS (pheochromocytoma of the adrenal gland scaled score) system, composed of twelve different histological features (7). This system has been frequently used for discerning malignancy in PHEOs, and PASS of ≧4 was proposed to be malignant or biologically more aggressive than those with PASS < 4 (7). However, it is also true that there have been controversies regarding the application of this system into the differential diagnosis between benign and malignant PHEOs including its reproducibility (7). Kimura et al. subsequently proposed another system to access malignant potential of PHEOs, *i.e.*, GAPP (grading system for pheochromocytoma and paraganglioma) (8). This system used both histological and clinical parameters including histological patterns, cellularity, comedo-type necrosis, capsular/vascular invasion, proliferative index (Ki-67) and catecholamine type. According to the GAPP system, PEHOs were subclassified into well-differentiated (WD), moderately differentiated (MD), or poorly differentiated tumor (PD), and this classification has been then used widely.

The tumor microenvironment (TME) has been reported to play pivotal roles in many human malignancies in tumorigenesis, progression, and metastasis and recently also explored as therapeutic targets (9). Histopathologically, TME is well known to be composed of a number of cellular components including inflammatory cells, blood vessels, fibroblasts, and others (10, 11). Of those components above, both T lymphocytes and macrophages have been generally considered to play pivotal roles in biological features of the patients (12, 13). In benign adrenocortical tumors, we previously demonstrated that cortisol-producing adenomas harbored higher immune cell infiltration and angiogenic markers than other hormone-producing adenomas (14). In adrenocortical carcinomas, tumor infiltrating T cells were also reported to be correlated with better overall survival (15). However, little has been known on TME in PHEOs at this juncture even in contrast to adrenocortical neoplasms above, although aggressive PHEOs were reported to harbor lower number of S100 positive sustentacular cells and increased angiogenesis (16–18). Therefore, in this study, we explored various TME relevant markers (CD4, CD8, CD68, CD163, and S100p), angiogenic markers (CD31, intratumoral hemorrhage area) and catecholamine-synthesizing enzymes (TH, DDC, and PNMT) of the tumors and compared the results with GAPP and PASS scoring systems, other clinicopathological factors of individual cases of 39 PHEOs in order to explore the possible correlation between the status of catecholamine production and TME and angiogenesis.

## Materials and Methods

### Pheochromocytoma Cases

We studied 39 adrenal PHEO patients operated at Tohoku University Hospital, Sendai, Japan from 2012 to 2019. Following the evaluation of 24 h urinary levels of metanephrine and normetanephrine, the diagnosis of PHEO was confirmed by their increased levels of at least three times than the normal range (2). We also applied PASS and GAPP in their histological diagnosis. Clinicopathological features of these patients examined were summarized in **Table 1**. Clinical information is not available due to the relatively new cases. Two patients experienced recurrence and one of them died. The present research protocol was approved by the Institutional Review Board (IRB) of Tohoku University School of Medicine (2018-1-669).

**Table 1:** Clinicopathological factors in PHEO patients.

Clinical factors	Average (range)
Age (years)	56 (39–79)
Gender: Female/Gender	25/14
BMI (kg/m^2^)	22.7 (15.48–31.49)
Tumor size (mm)	45.3 (20–100)
Tumor location: left/right	24/15
Duration of hypertension (years)	9.3 (0–25)
Plasma AD (ng/ml)	0.16 (0.01–0.742)
Plasma NAD (ng/ml)	1.8 (0.1–12)
24-h urine MN (mg/day)	1.7 (0.01–11)
24-h urine NMN (mg/day)	4.1 (0.01–26)
24-h urine AD (μg/day)	115.7 (1.9–510)
24-h urine NAD (μg/day)	775.9 (42.6–4200)

Gender and tumor location were described as the number of persons.

AD, adrenaline; NAD, noradrenaline; DA, dopamine; MN, metanephrine; NMN, normetanephrine; BMI, body mass index.

Normal range reference: plasma AD: 0–0.17 ng/ml; plasma NAD, 0.15–0.57 ng/ml; 24-h urine adrenaline, 1.1–22.5 mcg/day; 24-h urine noradrenaline, 29.2–118.0 mcg/day; 24-h urine metanephrine, 0.05–0.20 mg/day; 24-h urine normetanephrine, 0.10–0.28 mg/day.

### Immunohistochemistry and Its Evaluation

**Figure 1:**
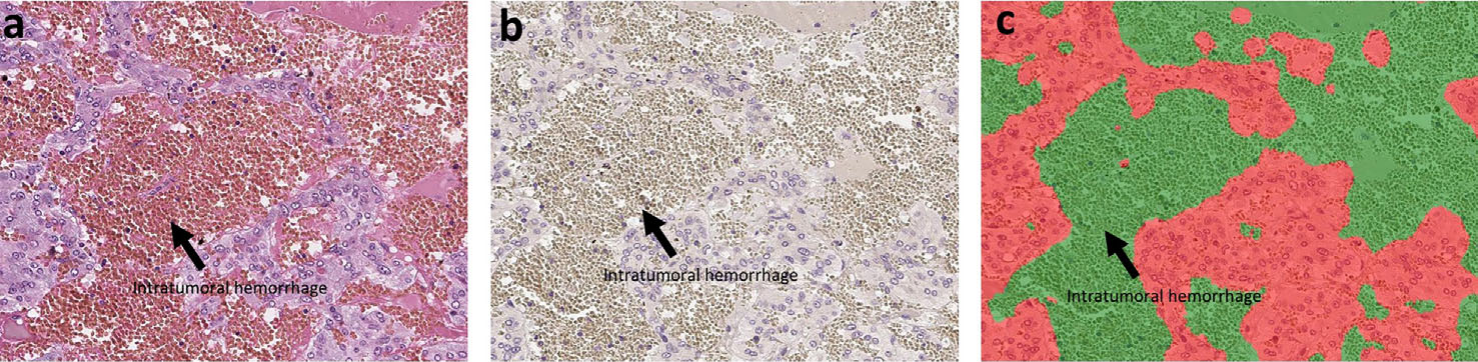
The analysis of intratumoral hemorrhage area. H&E stained section and hematoxylin-stained section are present in **(A, B)**. There were several intratumoral hemorrhage areas with gray color in **(B)**. Because the hemorrhage is composed of red blood cells that did not have a nucleus, it cannot be stained by hematoxylin. We used classifier system in HALO software to recognize the normal tumor area (blue) and the intratumoral hemorrhage area (gray). After analysis, the software can divide the tissue into two colors: red for tumor and green for hemorrhage **(C)**. Finally, we can calculate the ratio of intratumoral hemorrhage area against the whole tumor area.

Hematoxylin and eosin (H&E) staining and immunohistochemistry (IHC) were both performed in the specimens fixed in 10% buffered formalin and embedded in paraffin. The protocols for individual IHC markers used in our present study were summarized in **Table 2**. All the H&E and IHC sections were digitally scanned by Image Scope AT2 (Leica, Wetzler, Germany). Nuclear immunoreactivity of CD4, CD8, CD68, CD163, and S100p were all evaluated by the percentage of positive cells in tumoral parenchyma by manual analysis (19–22). Ki-67 labeling index was evaluated after identifying hot spot of the whole tumor (23). Microvascular density (MVD) was evaluated by counting the number of CD31-positive vessels within 0.75 mm^2^ and highest expressed tumor area (24). Immunoreactivity of catecholamine-synthesizing enzymes including tyrosine hydroxylase (TH), dopa-decarboxylase (DDC), and phentolamine-N-methyltransferase (PNMT) was all digitally and quantitatively evaluated by using “HALO™ CytoNuclear ver.1.5” (Indica Laboratories, Corrales, NM, USA) image analysis (25, 26). The positive cells were tentatively classified into the following four categories based on the levels of their relative immunointensity: negative as “0”, weak as “+1”, moderate as “+2”, and strong as “+3”. H-score was subsequently calculated based on the following formula; Σ (Number of the individual gradients of the positive cells X Score 1+, 2+, 3+)/Total cells) X100 (27–29). The evaluation of intratumoral hemorrhage area was also performed by using HALO software above according to the classifier system on H&E stained tissue sections (25). Under this system, tumor areas were tentatively classified into two portions: tumor and intratumoral hemorrhage areas (**Figure 1**). Hemorrhage in the central vein and ubiquitous necrosis were both carefully excluded in this analysis. The final value of intratumoral hemorrhage was the ratio of intratumoral hemorrhage against the whole tumor area.

**Table 2:** IHC protocols.

Antibody	Host	Clone	Dilution	Antigen retrieval	Source
**CD4**	Rabbit	EPR6855	1:400	AC	Abcam
**CD8**	Mouse	M7103	1:50	AC	Dako
**CD68**	Mouse	M0876	1:100	Trypsin	Dako
**CD163**	Mouse	M3527	1:600	AC	Dako
**S100p**	Rabbit	–	1:9,000	–	Dako
**CD31**	Mouse	M0823	1:100	AC	Dako
**Ki67**	Mouse	M7240	1:100	AC	Dako
**TH**	Mouse	T1299	1:2,000	MW	Sigma
**DDC**	Rabbit	AB136	1:500	–	Millipore
**PNMT**	Mouse	Ab119784	1:2,000	–	Abcam

AC, autoclave 121°C 5 min, pH = 6 buffer; MW, microwave 500 W 20 min, pH = 6 buffer.

### Statistical Analysis

We analyzed the correlations among TME relevant markers, angiogenic markers, catecholamine-synthesizing enzymes, histopathological score, and clinicopathological factors by *Spearman’s test*. The differences of TME relevant markers, angiogenic markers, and catecholamine-synthesizing enzymes in individual PASS and GAPP factors were all analyzed using *Mann*–*Whitney’s test*. We defined the significance as P-value <0.05. All the tests were analyzed using the software “JMP Pro ver. 14.0.0”.

## Results

### Comparison of PHEOs Between PASS ≧4 and PASS <4

**Figure 2:**
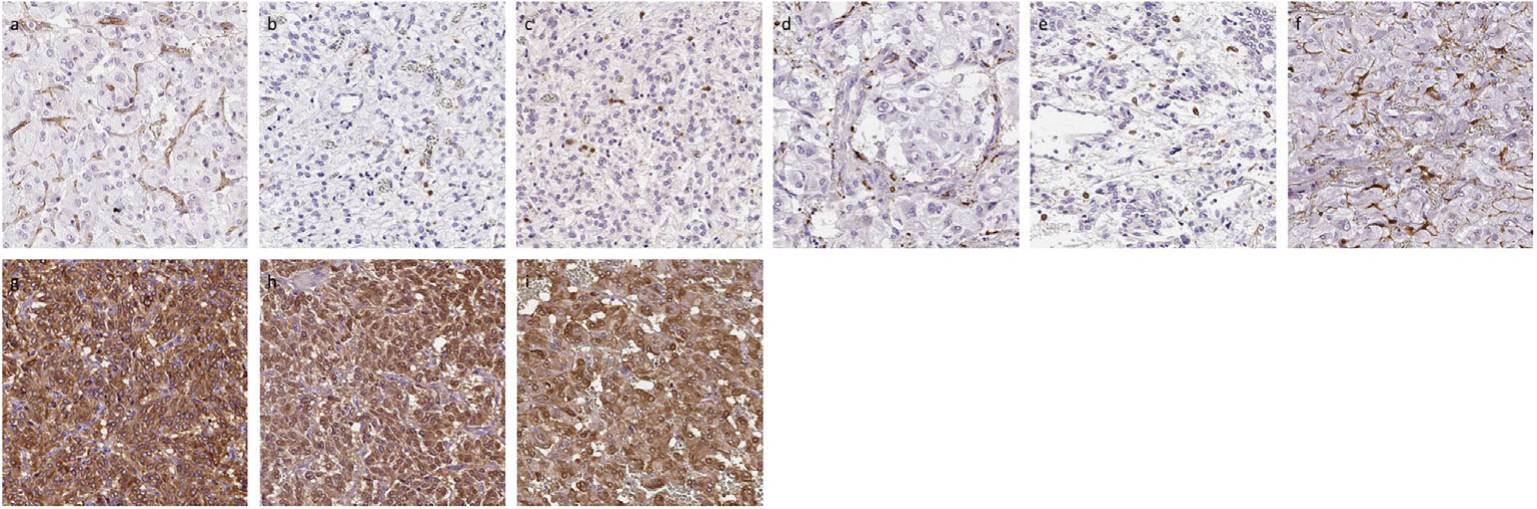
Representative IHC images of PHEOs. **(A)** CD31, **(B)** CD4, **(C)** CD8, **(D)** CD68, **(E)** CD163, **(F)** S100p, **(G)** TH, **(H)** DDC, **(I)** PNMT.

**Figure 3:**
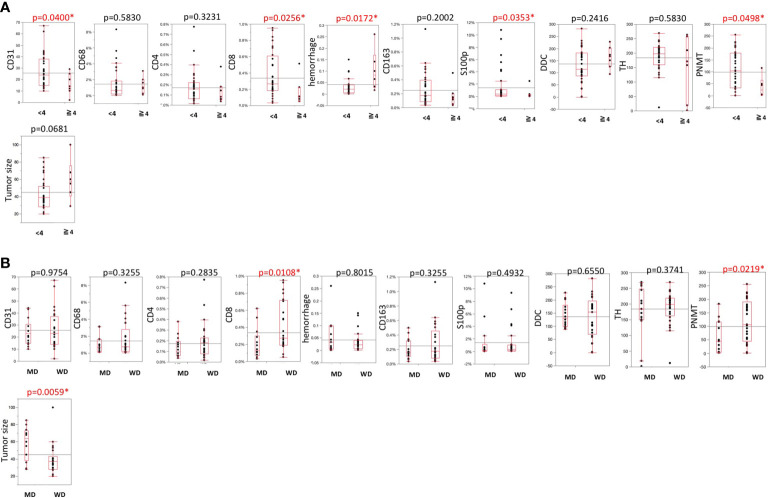
**(A)** Comparisons of TME-relevant markers, angiogenic marker, and catecholamine-synthesizing enzymes between PASS <4 and PASS ≧4. **(B)** Comparisons of TME-relevant markers, angiogenic marker, and catecholamine-synthesizing enzymes between WD and MD according to the GAPP system. All of the comparisons are presented **Figure 3**. P value was on the head of each factor.

Representative images were illustrated in **Figure 2**. Results were summarized in **Figure 3**. CD31-positive vessels (p = 0.0400), CD8 positive cells (p = 0.0256), S100p positive cells (p = 0.0353), and PNMT immunoreactivity (p = 0.0498) were all significantly higher in PHEOs with PASS <4 than those with PASS ≧4. The ratio of intratumoral hemorrhage was significantly higher in PHEOs with PASS ≧4 than PASS <4 (p = 0.0172).

### Comparison Between Well and Moderately Differentiated PHEOs According to GAPP Analysis

Analysis based on GAPP scores revealed as follows: 26 well-differentiated (WD), 13 moderately differentiated, and no poorly differentiated PEHOs (**Figure 3**). Both PNMT immunoreactivity (p = 0.0219) and CD8 positive cells (p = 0.0108) were significantly higher in WD- than MD-PHEOs. Tumor size was significantly smaller in WD- than MD-PHEOs (p = 0.0059). No other significant correlations were detected.

#### Comparison of TME, Angiogenesis, and Catecholamine-Synthesizing Enzymes With Results of PASS and GAPP Factors in Individual PHEOs

The histopathological factors of PASS and GAPP scoring systems were summarized in **Table 3**. Among individual factors of the PASS systems, the following were the numbers of positive individual cases per each factor: large nests of cells or diffuse growth in 10% of tumor: six; necrosis: three; high cellularity: four; cellular monotony: two; presence of spindle shaped tumor cells: two; increased mitotic figure (> 3 per 10 high power fields): zero; atypical mitosis: one; extension of tumor into adjacent fat: three; vascular invasion: 13; capsular invasion: five cases; profound nuclear pleomorphism: five; nuclear hyperchromasia: three (**Supplemental File 1**).

**Table 3:** PASS and GAPP systems.

PASS factors		Score
Large nests or diffuse growth (>10% of tumor volume)		2
Necrosis		2
High cellularity		2
Cellular monotony		2
Presence of spindle-shamed tumor cells		2
Atypical mitotic figures		2
Greater than 3 mitotic figures		2
Extension of tumor into adjacent fat		2
Vascular invasion		1
Capsular invasion		1
Profound nuclear pleomorphism		1
Nuclear hyperchromasia		1
**Total**		**20**
**GAPP factors**	**Score**	
**Histological pattern:**	
Zellballen		0	
Large and irregular cell nest		1
Pseudorosette		1	
**Cellularity:**	
Low (<150 cells/U)		0	
Moderate (150–250 cells/U)		1	
High (>250 cells/U)		2	
**Comedo necrosis:**	
Absence		0	
Presence		2	
**Vascular or capsular invasion:**	
Absence		0	
Presence		1	
**Ki-67 labeling index:**	
<1%		0
1–3%		1
>3%		2
**Catecholamine type:**	
Epinephrine type (E or E + NE)		0
Norepinephrine type (NE or NE + AD)		1
Non-functioning type		0
**Total maximum score**		**10**

U, number of tumor cells under ×400 magnification; E, epinephrine; NE, norepinephrine.

Among the six GAPP factors examined in 39 PHEOs in our present study, 13 demonstrated abnormal histological features, 11 low cellularity (<150/HPF), 19 moderate cellularity (150–250/HPF) and nine high cellularity (>250/HPF), one comedo-necrosis, 13 capsule or vascular invasion, 26 low Ki-67 LI (<1%), nine moderate Ki-67 LI (1–3%) and four high Ki-67 LI (>3%), 10 norepinephrine predominant secretion (**Supplemental File 2**). CD68 positive cells were significantly higher in PHEOs with normal histological patterns than those with abnormal patterns (p = 0.0370). DDC in tumor cells was significantly more abundant in PHEOs with capsule or vascular invasion than those without (p = 0.0172). TH in tumor cells was significantly more abundant in PHEOs with normal histological patterns than PHEOs with abnormal patterns (p = 0.0236).

#### Correlations Among TME Relevant Markers, Angiogenic Markers, and Catecholamine-Synthesizing Enzymes

Results were summarized in **Table 4**. CD163 positive cells were significantly positively correlated with CD4 (p = 0.0405) and CD8 (p = 0.0032) positive cells in the tumor. The ratio of intratumoral hemorrhage areas was inversely correlated with CD31-positive vessels (p = 0.0785). CD8 positive cells were significantly inversely correlated with GAPP score (p = 0.0018). CD68 positive cells were positively correlated with TH (p = 0.0901) and DDC (p = 0.0168) immunoreactivity in tumor cells. S100p positive cells were significantly inversely correlated with PASS (p = 0.0205) and GAPP (p = 0.0217) scores. Hemorrhage score was significantly positively correlated with PASS score (p = 0.0059). DDC was positively correlated with PASS score (p = 0.0365) but PNMT was inversely significantly correlated with PASS (p = 0.0632) and GAPP score (p = 0.0003).

**Table 4:** Correlations among TME-relevant, angiogenetic markers, and catecholamine-synthesizing enzymes.

	CD31	CD4	CD8	CD68	CD163	S100	Ki-67	TH	DDC	PNMT	PASS score	GAPP score
**CD31**								ρ = 0.0013 p = 0.9938	ρ = −0.0277 p = 0.8688	ρ = −0.0737 p = 0.6647	ρ = −0.1980 p = 0.2333	ρ = −0.0119 p = 0.9436
**CD4**	ρ = 0.1069 p = 0.5230							ρ = −0.1340 p = 0.4160	ρ = −0.1138 p = 0.4905	ρ = 0.2804 p = 0.0881	ρ = −0.1894 p = 0.2482	ρ = −0.1110 p = 0.5013
**CD8**	ρ = −0.0650 p = 0.6982	ρ = 0.0395 p = 0.8114						ρ = −0.0312 p = 0.8505	ρ = −0.1795 p = 0.2743	ρ = 0.1672 p = 0.3156	ρ = −0.1714 p = 0.2969	ρ = −0.4839 p = 0.0018*
**CD68**	ρ = −0.0629 p = 0.7077	ρ = 0.0759 p = 0.6460	ρ = 0.1822 p = 0.2669					ρ = 0.2751 p = 0.0901	ρ = 0.3806 p = 0.0168*	ρ = 0.2041 p = 0.2191	ρ = 0.0612 p = 0.7113	ρ = −0.1848 p = 0.2600
**CD163**	ρ = 0.0460 p = 0.7839	ρ = 0.3296 p = 0.0405*	ρ = 0.4604 p = 0.0032*	ρ = −0.0200 p = 0.9036				ρ = 0.0435 p = 0.7925	ρ = 0.0022 p = 0.9893	ρ = 0.0058 p = 0.9724	ρ = −0.1593 p = 0.3327	ρ = −0.1668 p = 0.3103
**S100p**	ρ = 0.1404 p = 0.4005	ρ = 0.2008 p = 0.2203	ρ = 0.2642 p = 0.1041	ρ = 0.0277 p = 0.8669	ρ = 0.1054 p = 0.5232			ρ = −0.0108 p = 0.9478	ρ = −0.0420 p = 0.7996	ρ = 0.2185 p = 0.1875	ρ = −0.3699 p = 0.0205*	ρ = −0.3665 p = 0.0217*
**Ki-67**	ρ = −0.0841 p = 0.6206	ρ = 0.1014 p = 0.5445	ρ = 0.0039 p = 0.9813	ρ = 0.0863 p = 0.6063	ρ = 0.1623 p = 0.3304	ρ = -0.2206 p = 0.1832		ρ = 0.1977 p = 0.2341	ρ = −0.0012 p = 0.9943	ρ = 0.0028 p = 0.986 7	ρ = 0.2319 p = 0.1612	ρ = 0.2909 p = 0.0764
**Hemorrhage**	ρ = −0.2955 p = 0.0758	ρ = −0.1605 p = 0.3358	ρ = 0.0630 p = 0.7070	ρ = 0.0667 p = 0.6909	ρ = 0.0454 p = 0.7865	ρ = -0.1501 p = 0.3684	ρ = -0.1555 p = 0.3581	ρ = 0.0055 p = 0.9740	ρ = 0.1228 p = 0.4625	ρ = −0.0125 p = 0.9417	ρ = 0.4385 p = 0.0059*	ρ = 0.0575 p = 0.7317
**PASS score**								ρ = −0.1492 p = 0.3648	ρ = 0.3376 p = 0.0356*	ρ = −0.3044 p = 0.0632		
**GAPP score**								ρ = −0.1598 p = 0.3313	ρ = 0.1808 p = 0.2708	ρ = −0.5580 p = 0.0003*		

*p < 0.05.

### Correlations of TME Relevant Markers, Angiogenic Markers With Clinicopathological Factors in PHEOs

The correlations among TME relevant markers, angiogenic markers and catecholamine-synthesizing enzymes and clinicopathological factors were summarized in **Table 5**. Plasma adrenaline levels were significantly positively correlated with CD8 (p = 0.0521) and CD68 (p = 0.0962) positive cells and significantly inversely correlated with GAPP score (p = 0.0191). Urinary AD was inversely correlated with GAPP score (p = 0.0605). PNMT expression was significantly inversely correlated with urinary NAD (p = 0.0318). Tumor size was significantly inversely correlate with CD8 (p = 0.0257), CD68 (p = 0.0457), S100 (p = 0.0035), PNMT (p = 0.0009) and positively correlated with PASS (p = 0.0710) and GAPP score (p = 0.0006).

**Table 5:** Correlations among TME-relevant markers, plasma, and urine catecholamines and clinicopathological factors.

	Plasma AD	Plasma NAD	Urine AD	Urine NAD	Tumor size
**CD31**	ρ = 0.0415 p = 0.8075	ρ = 0.1527 p = 0.3668	ρ =−0.0875 p = 0.6119	ρ = 0.1179 p = 0.4935	ρ = −0.0226 p = 0.8957
**CD4**	ρ = −0.0677 p = 0.6863	ρ = −0.0748 p = 0.6556	ρ = 0.0937 p = 0.5814	ρ = 0.0254 p = 0.8815	ρ = −0.0235 p = 0.8901
**CD8**	ρ = 0.3175 p = 0.0521	ρ = −0.0718 p = 0.6683	ρ = 0.2197 p = 0.1914	ρ = −0.1190 p = 0.4829	ρ = −0.3665 p = 0.0257*
**CD68**	ρ = 0.2738 p = 0.0962	ρ = 0.1993 p = 0.2303	ρ = 0.1496 p = 0.3768	ρ = −0.1107 p = 0.5142	ρ = −0.3305 p = 0.0457*
**CD163**	ρ = 0.0359 p = 0.8304	ρ = 0.0638 p = 0.7035	ρ = −0.0211 p = 0.9013	ρ = 0.0318 p = 0.8519	ρ = 0.0638 p = 0.7077
**S100p**	ρ = 0.0141 p = 0.9331	ρ = 0.0464 p = 0.7820	ρ = 0.0598 p = 0.7254	ρ = 0.0537 p = 0.7523	ρ = −0.4676 p = 0.0035*
**Ki-67**	ρ = −0.0145 p = 0.9322	ρ = −0.1544 p = 0.3616	ρ = 0.0049 p = 0.9774	ρ = −0.2530 p = 0.1365	ρ = −0.0181 p = 0.9168
**Hemorrhage**	ρ = 0.0425 p = 0.8028	ρ = −0.2718 p = 0.1037	ρ = 0.0644 p = 0.7091	ρ = −0.2752 p = 0.1043	ρ = 0.0595 p = 0.7301
**TH**	ρ = −0.0956 p = 0.5679	ρ = 0.1085 p = 0.5169	ρ = −0.2388 p = 0.1547	ρ = −0.1183 p = 0.4856	ρ = −0.3171 p = 0.0558
**DDC**	ρ = −0.1402 p = 0.4010	ρ = 0.1288 p = 0.4408	ρ = −0.1721 p = 0.3083	ρ = 0.0782 p = 0.6453	ρ = −0.1125 p = 0.5075
**PNMT**	ρ = 0.2564 p = 0.1256	ρ = −0.2727 p = 0.1025	ρ = 0.3052 p = 0.0703	ρ = −0.3586 p = 0.0318*	ρ = −0.5290 p = 0.0009*
**PASS score**	ρ = −0.0269 p = 0.8726	ρ = −0.1252 p = 0.4539	ρ = −0.0056 p = 0.9739	ρ = −0.1274 p = 0.4523	ρ = 0.2961 p = 0.0710
**GAPP score**	ρ = −0.3787 p = 0.0191*	ρ = 0.0396 p = 0.8136	ρ = −0.3115 p = 0.0605	ρ = 0.0977 p = 0.5653	ρ = 0.5316 p = 0.0006*

*:p < 0.05.

## Discussion

TME including tumor infiltrating lymphocytes (TILs), tumor-associated macrophages (TAMs) as well as angiogenesis has all been reported to play pivotal roles in not only clinical but also therapeutic outcomes in various human malignancies. However, TME and other relevant phenomena have not necessarily been well studied in adrenal medullary tumor including PHEOs although decreased S100 was detected in benign PHEOs (17, 18, 30). In our present study, we did compare the status of S100 positive cells with results of PASS analysis and obtained similar findings reported above (7). Both PASS and GAPP scoring systems have been used relatively widely for surgical pathologists to evaluate the malignant potential of the patients with PHEOs. PASS score could be obtained only from routinely available histopathological findings and tumor with PASS of four or more than four score was considered malignant (7). However, relatively marked inter- and intra-observer variations have been reported in application of PASS scoring system (31). Therefore, we also employed GAPP system as well, which included not only morphological features but also catecholamine secretary phenotypes and Ki-67 labeling index (8), in order to further explore the malignant potential of PHEOs in a more precise fashion (32).

In this study, we firstly evaluated the details of TILs. The status of CD8^+^ TILs has been generally reported to be correlated with better prognosis (33–35). Our results did demonstrate that CD8^+^ TILs could play pivotal roles in possible biological behavior of PHEOs and its abundance could also represent relatively benign tumors as reported in other human malignancies (33–35). However, it awaits further investigations for clarification.

Dendritic cells activated anti-tumor CD8^+^ T lymphocytes (CTLs) by presenting tumor antigen and then effector CTLs executed its roles of eliminating tumor cells (36). CTLs can induce an apoptosis of tumor cell by FasL pathway and can also release some cytokines to induce cytotoxicity in tumor cells (37). In our present study, we demonstrated an inverse correlation between CTLs and tumor size, which indicated that CTLs could suppress the expansion of tumor. However, CTLs were also suppressed in malignant PHEOs possibly by excessive catecholamine derived from tumor cells, themselves (38). Catecholamine was reported to suppress T lymphocyte such as T-helper 1/2, cytotoxic CD8^+^T-lymphocytes and NK cells (39–41). Both epinephrine and norepinephrine were also reported to exert inhibitory effects on T-cell proliferation due to chronic mild stress in mice (42). Therefore, results of our present study also indicated that CTLs could be suppressed by excessive catecholamine produced by malignant PHEOs. In addition, in our present study, CD68^+^ cells were significantly higher in PHEOs with normal histological patterns and negatively correlated with tumor size. Intratumoral CD163 positive cells were significantly positively correlated with the status of intratumoral CD4 and CD8 infiltrating cells. Macrophages constituted the great majority of cellular components in tumor stroma with noticeable plasticity and could be further subclassified into M1 and M2 phenotypes (37). M1 macrophages were classically defined as activated macrophages and reported to play pro-inflammation roles resulting in overall antitumor effects (37, 43, 44). On the other hand, M2 macrophages were reported to have opposite roles toward M1 macrophages resulting in pro-tumor effects (37, 44, 45). In addition, the differentiation to M1 or M2 macrophages was reported to be dependent on cytokines (44). For instance, differentiation to M1 macrophages could be induced by IFN-*γ*, TNF-α, IL-8, and IL-12 secreted from CTLs. M2 macrophages were reported to be induced by STAT3, IL4, and IL-10 and to subsequently suppress T cell function. In addition, M2 macrophages were the representative subtypes of macrophages in TME of most human malignancies including breast, urinary bladder, and prostate carcinoma (46, 47). Results of our present study did demonstrate that TAMs in PHEOs could be predominantly reprogrammed into CD68^+^M1 rather than CD163+M2 macrophages, which exerted potential anti-tumoral effects by secreting different cytokines (44, 48). However further investigations are required to clarify the details.

Recently, TILs have been also reported to be associated with overall survival and recurrence-free survival of the patients with adrenocortical carcinomas (ACCs). However, of particular interest, the difference between ACCs and PHEOs was that not only CD8^+^ but also CD4^+^ T lymphocytes were related to relatively better clinical outcome in the patients with ACCs (15). CD4^+^ T cells are helper T cells, stimulating CTLs activities (37). In addition, ACCs were also relatively frequently detected in childhood and increased TILs were reported in those young ACCs patients than adults (49). The most notable difference between ACCs and PHEOs was considered the effects of different hormones on TILs. The great majority of PHEOs were characterized by catecholamine excess, which could suppress T-cell activity. On the other hand, ACC was characterized by the excessive steroids including relatively abundant precursor steroids and steroid hormones could in general hamper anti-tumor roles of T-cells (50–53). Therefore, tumor microenvironment could be influenced by those hormones secreted by tumor cells, but it awaits further investigations for clarification.

Angiogenesis is indispensable and pivotal for tumor growth, invasion and metastasis (54). Hypoxia has been reported one of the most pivotal causes of angiogenesis in PHEOs (55). Increased endothelial growth factor (VEGF) has been reported in malignant PHEOs and to be associated with hypoxia inducible factor (HIF) (56–59). PHEOs have been well known to be associated with increased angiogenesis due to pseudohypoxic signaling pathway (60). Among the factors involved in this pathway, *SDHXs* were the most susceptive genes (60). Pseudohypoxic subtype has been also reported to be associated with aggressive biological behavior (60). The pathophysiological features of this particular phenotype could therefore promote the neoplastic angiogenesis. In our present study, intratumoral hemorrhage was abundantly detected in histologically high-graded tumors, which could also result in relatively low CD31 status in histologically low-graded tumors. However, genetic testing was not performed as a routine in our institution and this could also represent the limitation of our present study.

In general, norepinephrine-producing PHEOs were reported to accompany PNMT deficiency which is considered to be less differentiated than epinephrine-producing PHEOs (7, 17). Dopamine-secreting PHEOs were also considered less differentiated or immature and had a high prevalence of malignancy (59, 60). Results of our present study were also consistent with those findings above. The absence of TH in some PHEOs in our present study could not only result in non-functional nature of the tumors but also in increased aggressive biological behavior (61, 62). Therefore, the abnormal elevation of plasma dopamine or the absence of TH immunoreactivity in PHEOs could indicate more aggressive biological behavior of the tumor.

It is true that there are several limitations in our present study. The number of the cases examined was relatively small. In addition, the cases examined were relatively new cases which could make it difficult to obtain the long-term clinical outcome of the patients. Genetic testing was not performed routinely in our hospital. Therefore, those above were considered limitations of this study. Further investigations are also required to clarify the detailed mechanisms of interaction between TME and tumor cells in PHEOs.

In summary, we firstly presented detailed features of TILs and TAMs in PHEOs. CD8 and CD68 could also serve as biomarkers of well-differentiated or histologically low-scored PHEOs. Benign PHEOs harbored smaller sized tumor than malignant ones, also related both TILs and TAMs. Poorly differentiated PHEOs had increased incidence of intratumoral hemorrhage and the absence of TH in tumor cells.

## Data Availability Statement

The raw data supporting the conclusions of this article will be made available by the authors, without undue reservation.

## Ethics Statement

The studies involving human participants were reviewed and approved by the Institutional Review Board of Tohoku University School of Medicine (2018-1-669). The patients/participants provided their written informed consent to participate in this study.

## Author Contributions

XG, YY, and AP contributed to staining and evaluation of H&E and IHC sections. YT, KO, YO, and RM contributed to the collection of clinical data from Tohoku University hospital. YN, FS, and HS supervised all of the present study. All authors contributed to the article and approved the submitted version.

## Funding

XG was supported by Global Hagi scholarship by Tohoku University. This work was supported by a Health Labour Sciences Research Grant (No. H29-Nanji-Ippan-046).

## Conflict of Interest

The authors declare that the research was conducted in the absence of any commercial or financial relationships that could be construed as a potential conflict of interest.
