# Supplementary material for: Histopathological Analysis of Tumor Microenvironment and Angiogenesis in Pheochromocytoma
Source: Front Endocrinol (Lausanne). 2020 Nov 10;11:587779. doi: 10.3389/fendo.2020.587779 (PMC7685215; doi:10.3389/fendo.2020.587779)
Supplement: Supplementary file 1 [file Presentation_1.pptx]

## Slide 1
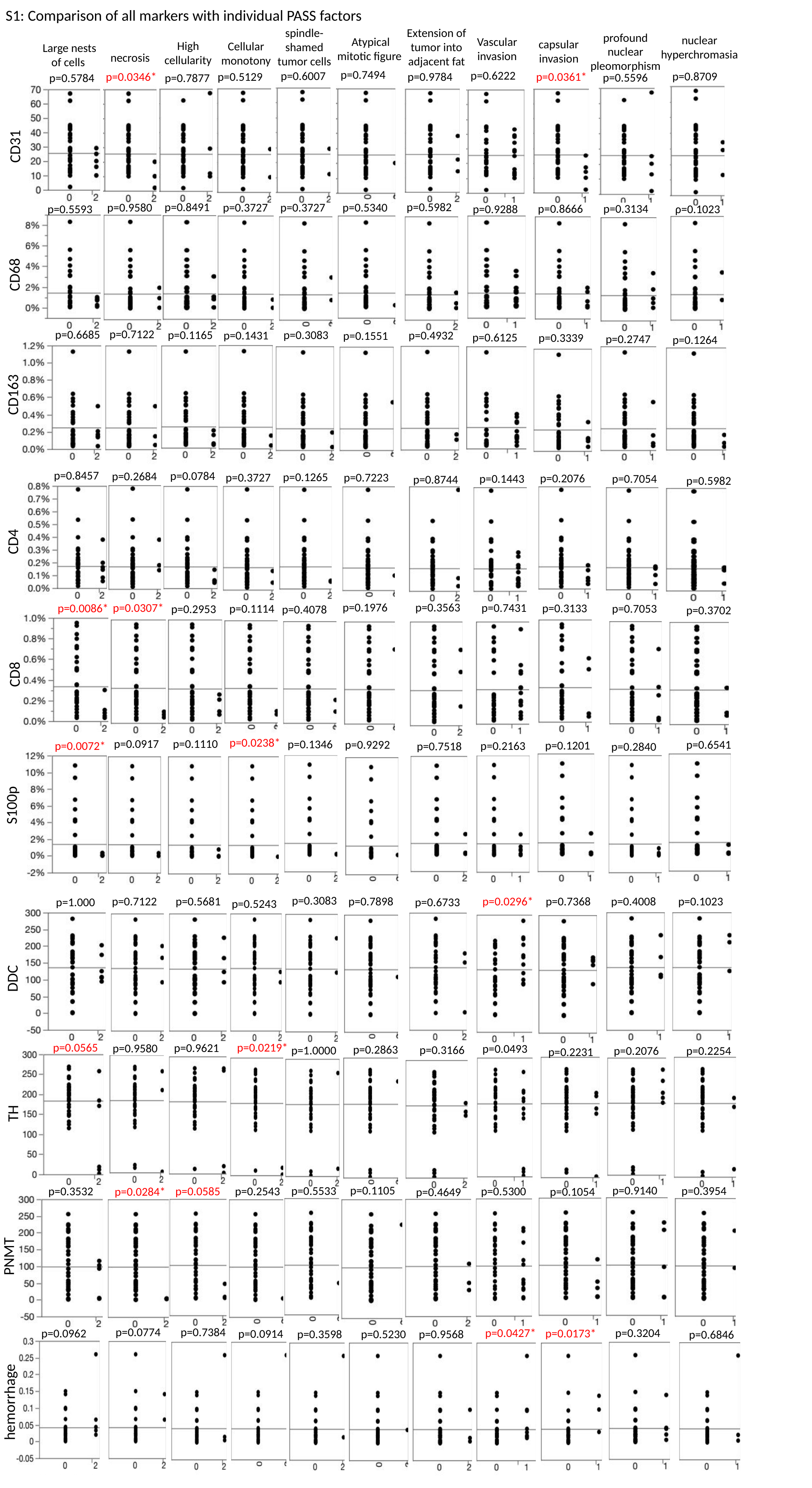

S1: Comparison of all markers with individual PASS factors
spindle-shamed tumor cells
Extension of tumor into adjacent fat
profound nuclear pleomorphism
nuclear hyperchromasia
Atypical mitotic figure
Vascular invasion
capsular invasion
High cellularity
Cellular
monotony
Large nests of cells
necrosis
p=0.7494
p=0.6222
p=0.6007
p=0.8709
p=0.0346*
p=0.9784
p=0.0361*
p=0.5129
p=0.5596
p=0.5784
p=0.7877
CD31
p=0.8491
p=0.5982
p=0.3727
p=0.9580
p=0.3727
p=0.5340
p=0.3134
p=0.8666
ρ=0.1023
p=0.5593
p=0.9288
CD68
p=0.7122
p=0.6685
p=0.1165
p=0.3083
p=0.1431
p=0.4932
p=0.1551
p=0.6125
p=0.3339
p=0.2747
p=0.1264
CD163
p=0.8457
p=0.0784
p=0.2684
p=0.1265
p=0.3727
p=0.7223
p=0.2076
p=0.1443
p=0.7054
p=0.8744
p=0.5982
CD4
p=0.3563
p=0.1976
p=0.0307*
p=0.7431
p=0.0086*
p=0.3133
p=0.1114
p=0.7053
p=0.2953
p=0.4078
p=0.3702
CD8
p=0.0238*
p=0.1110
p=0.0917
p=0.1346
p=0.9292
p=0.6541
p=0.1201
p=0.0072*
p=0.2163
p=0.7518
p=0.2840
S100p
p=0.3083
p=0.7898
p=0.7122
p=0.5681
p=0.0296*
p=0.7368
p=0.4008
p=0.1023
p=1.000
p=0.6733
p=0.5243
DDC
p=0.0219*
p=0.0565
p=0.9621
p=0.9580
p=0.0493
p=0.2863
p=0.3166
p=1.0000
p=0.2254
p=0.2076
p=0.2231
TH
p=0.9140
p=0.1105
p=0.5533
p=0.3954
p=0.2543
p=0.3532
p=0.5300
p=0.0585
p=0.1054
p=0.0284*
p=0.4649
PNMT
p=0.7384
p=0.0774
p=0.0427*
p=0.3204
p=0.0962
p=0.0173*
p=0.0914
p=0.5230
p=0.3598
p=0.9568
p=0.6846
hemorrhage

## Slide 2
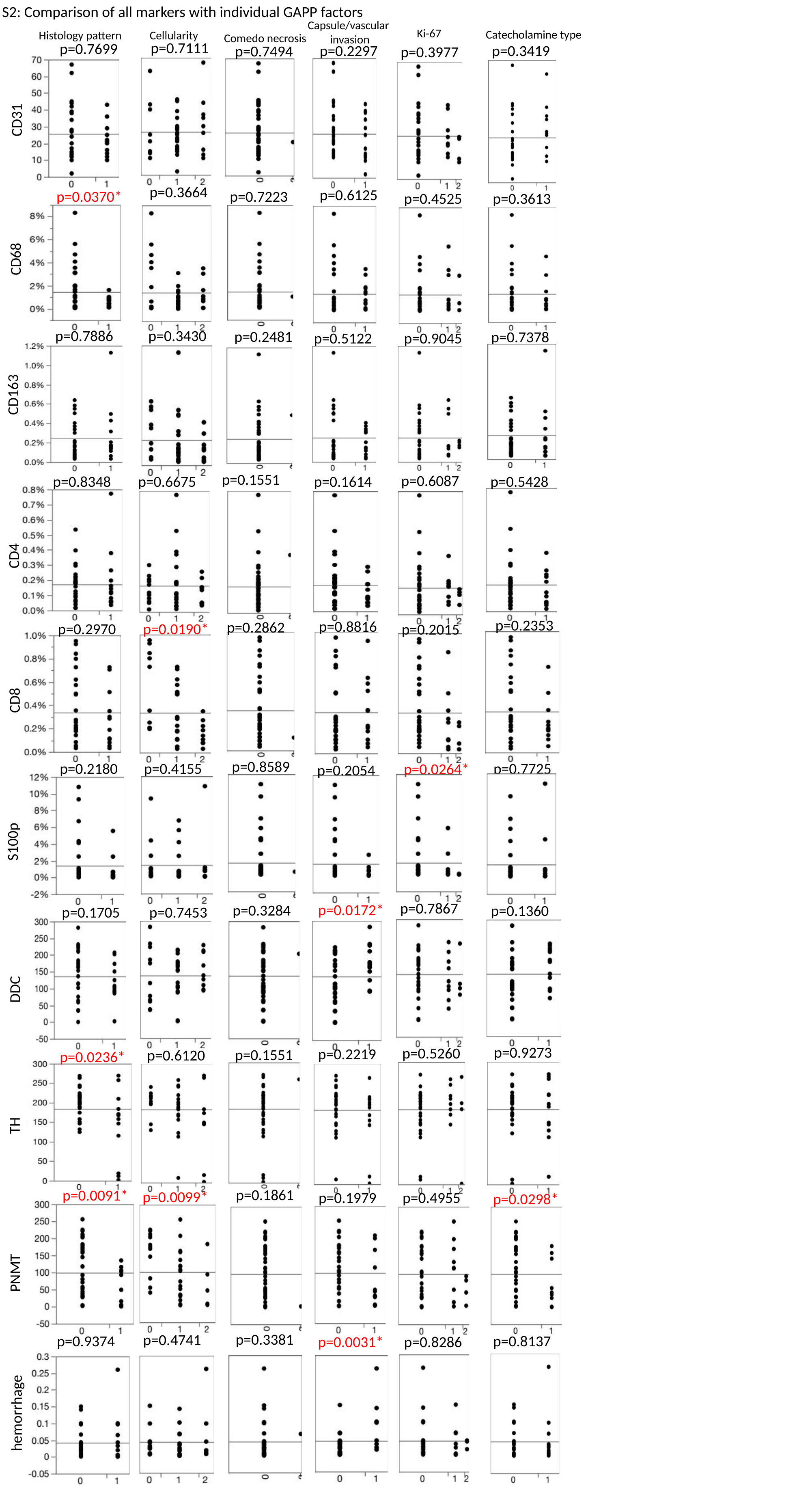

S2: Comparison of all markers with individual GAPP factors
Capsule/vascular
invasion
Ki-67
Catecholamine type
Cellularity
Histology pattern
Comedo necrosis
p=0.7111
p=0.7699
p=0.2297
p=0.3419
p=0.7494
p=0.3977
CD31
p=0.3664
p=0.6125
p=0.0370*
p=0.7223
p=0.4525
p=0.3613
CD68
p=0.7886
p=0.3430
p=0.2481
p=0.7378
p=0.9045
p=0.5122
CD163
p=0.1551
p=0.6087
p=0.8348
p=0.6675
p=0.1614
p=0.5428
CD4
p=0.2353
p=0.8816
p=0.2862
p=0.2970
p=0.0190*
p=0.2015
CD8
p=0.8589
p=0.4155
p=0.2180
p=0.7725
p=0.0264*
p=0.2054
S100p
p=0.7867
p=0.0172*
p=0.3284
p=0.1360
p=0.1705
p=0.7453
DDC
p=0.9273
p=0.5260
p=0.2219
p=0.1551
p=0.6120
p=0.0236*
TH
p=0.1861
p=0.0091*
p=0.0099*
p=0.1979
p=0.4955
p=0.0298*
PNMT
p=0.3381
p=0.4741
p=0.9374
p=0.8137
p=0.8286
p=0.0031*
hemorrhage
